# 519. Antibodies Produced by Children with Kawasaki Disease (KD) Consistently Recognize Antigen in KD Tissues from the US and Japan

**DOI:** 10.1093/ofid/ofae631.171

**Published:** 2025-01-29

**Authors:** Anne H Rowley, David Arrollo, Stanford Shulman, Masaru Terai, Kassandra Mercado, Kristine Wylie, Susan Baker

**Affiliations:** Northwestern University, Chicago, IL; Ann and Robert H Lurie Children's Hospital of Chicago, Chicago, Illinois; Ann & Robert H. Lurie Children's Hospital of Chicago, Chicago, Illinois; Chiba Kaihin Municipal Hospital, Chiba, Chiba, Japan; Ann and Robert H Lurie Children's Hospital of Chicago, Chicago, Illinois; Washington University in St Louis, St Louis, Missouri; Loyola University Chicago Stritch School of Medicine, Maywood, IL

## Abstract

**Background:**

We reported that synthetic versions of IgA antibodies present in inflamed arterial tissue from a patient with fatal KD (*JID 2004;190:856-65*) and from circulating IgA/IgG plasmablasts from 9/11 children with acute KD (*JID 2020;222:158-68*) identified antigen within intracytoplasmic inclusion bodies (ICI) in ciliated bronchial epithelial cells and macrophages in inflamed tissues of fatal KD cases. We performed additional studies to extend these findings.

**Figure 1.**
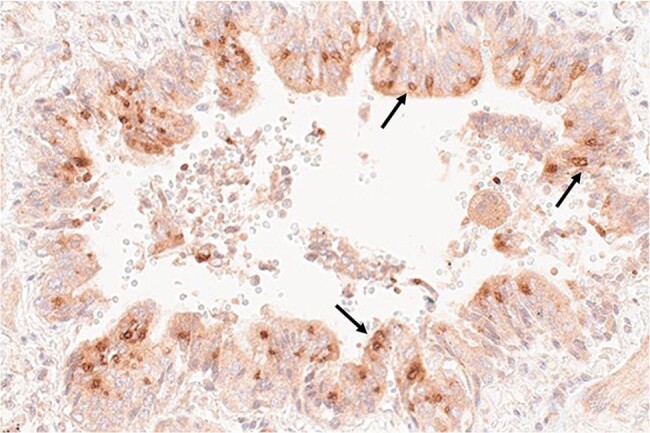
KD antigen in bronchus of a Japanese child with KD. Antigen (brown) detected by IHC using MAb KD7-1D3 resides in intracytoplasmic inclusion bodies (arrows) in bronchial epithelial cells.

**Methods:**

Monoclonal antibodies (MAbs) were produced from circulating plasmablasts from children with acute KD by cloning their immunoglobulin sequences into rabbit heavy chain and human light chain expression vectors and transfecting 293F cells. Immunohistochemistry (IHC) was performed on formalin-fixed, paraffin-embedded (FFPE) tissues from acute KD fatalities from the US and Japan.

**Figure 2.**
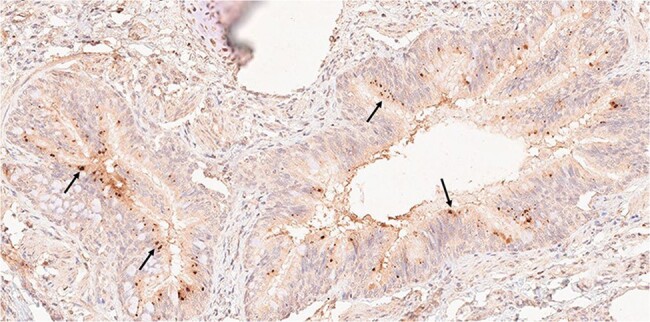
KD antigen in bronchus of a US child with KD. Antigen (brown) detected by IHC using MAb KD7-1D3 resides in intracytoplasmic inclusion bodies (arrows) in bronchial epithelial cells.

**Results:**

To date, we have produced ∼100 MAbs from circulating plasmablasts from 12 patients with acute KD. We tested selected MAbs for reactivity with the KD peptide we reported in *JID 2023; 228:412-21* (78 tested, with 34 (46%) positive) and/or tested them by IHC on KD tissues (62 tested, with 37 (60%) positive). At least one MAb from each of the 12 patients was positive by IHC, reacted with KD peptide, or both. IHC revealed antigen(s) in ICI in bronchial epithelium in all 10 US and 10 Japanese KD cases who died within one month after fever onset. Antigen was absent in infant controls who died of RSV, influenza, or congenital heart disease.

**Figure 3.**
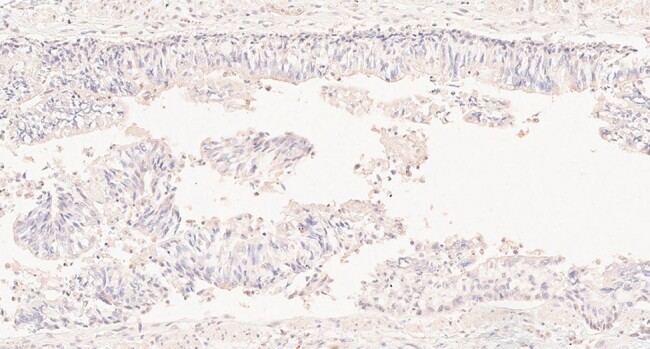
Bronchus from a child with RSV infection showing absence of staining. IHC using MAb KD7-1D3 shows negative results.

**Conclusion:**

The consistent detection of KD antigen(s) in fatal KD cases from the US and Japan targeted by MAbs from children with acute KD strongly supports the importance of the antigen(s) in KD pathogenesis. The presence of antigen(s) in bronchial epithelium strongly suggests that they derive from a single novel respiratory virus common to KD patients that can travel in macrophages to damage the coronary arteries. Because fatal cases are rare and FFPE is the tissue type available, the exact identification of the antigen(s) has been very difficult, although a peptide epitope/mimotope has been identified. Identifying the antigens targeted by KD MAbs currently offers the best opportunity for elucidating the etiologic agent of KD and should be a major focus of future KD research.

**Figure 4.**
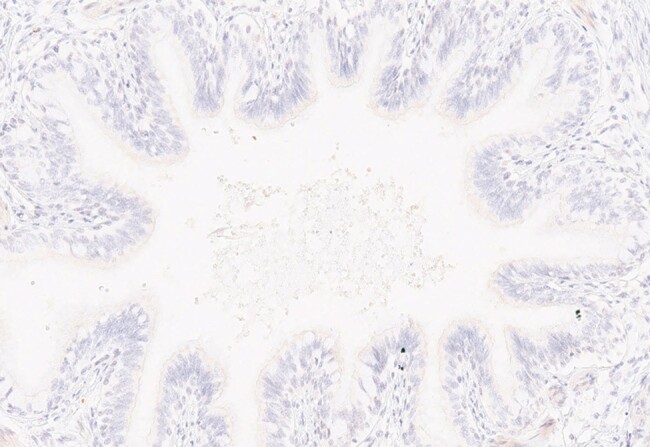
Bronchus from a child with complex congenital heart disease showing absence of staining.

IHC using MAb KD7-1D3 shows negative results.

**Disclosures:**

**All Authors**: No reported disclosures

